# Combination of Microwave-Assisted Girard Derivatization with Ionic Liquid Matrix for Sensitive MALDI-TOF MS Analysis of Human Serum N-Glycans

**DOI:** 10.1155/2018/7832987

**Published:** 2018-10-21

**Authors:** Hoa Thi Le, Kyu H. Park, Woong Jung, Hyung Soon Park, Tae Woo Kim

**Affiliations:** ^1^Graduate School of East-West Medical Science, Kyung Hee University, Yongin 17104, Republic of Korea; ^2^R&D Center, ASTA Inc., Suwon 16229, Republic of Korea; ^3^Department of Emergency Medicine, School of Medicine, Kyung Hee University, Seoul 02447, Republic of Korea

## Abstract

We developed a new method for MALDI-TOF MS detection of N-glycans derived from human serum. The synergistic combination of microwave-assisted Girard T derivatization, solid-phase extraction desalting, and an ionic liquid matrix (2, 5-dihydroxybenzoic acid/aniline) (GT-SPE-DHB/An) allowed of more sensitive N-glycans detection than a conventional ionic liquid matrix in MALDI-TOF MS. The superior sensitivity of our method was confirmed by the number of assigned N-glycans in 900–2,000 m/z range. Using our GT-SPE-DHB/An method, we were successfully able to assign 31 glycans. However, with the established method, i.e., DHB/An method, only 15 glycans were assigned. To the best of our knowledge, this GT-SPE-DHB/An method is the first to combine cationic derivatization of N-glycan and ionic liquid matrix for N-glycan analysis in MALDI-TOF MS.

## 1. Introduction

N-Linked glycans are involved in many biological processes such as protein folding, cell-cell interaction, and immune response [1, 2]. More specifically, N-glycosylation changes in the serum glycome are related to cancer and inflammation [3], and serum glycome profiling can serve as tumor biomarkers [4]. Therefore, N-glycan analysis in human serum is of central importance in biomarker discovery [5] and cancer diagnostics [6]. Despite their importance, however, the following characteristics of glycans make glycan analysis more challenging: heterogeneity of N-glycosylation [7] and absence of a unified database for glycome structure [8].

Glycan analysis based on matrix-assisted laser desorption/ionization- (MALDI-) time of flight (TOF) mass spectrometry (MS) has several advantages: low sample consumption, high sensitivity, and simple sample preparation. Nevertheless, MALDI-TOF MS analysis of glycans still has its own drawbacks. They generally stem from inherent low ionization efficiency of glycans [9], variability in crystallization [10], and high noise level of matrix material in minor-abundant glycan analysis [11].

**Scheme 1:**
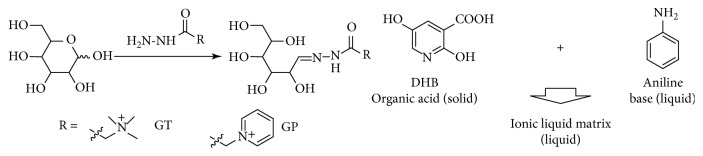
Glycan derivatization chemistry and ionic liquid matrix. (a) Hydrazide reaction of Girard reagents with a hexose. GT = Girard's reagent T (tetramethyl ammonium); GP = Girard's reagent P (pyridium). (b) ILM formation of 2, 5-dihydroxybenzoic acid and aniline.

To solve the problems, researchers have developed glycan derivatization methods and ionic liquid matrices (ILMs). Glycan derivatization includes reductive amination, Michael addition, hydrazide/alkoxy amino labeling, and (per)methylation/pertrimethylsilylation [12–14]. Especially, the hydrazide chemistry has been widely used for labeling reducing end of carbohydrate (Scheme 1). Girard's reagent T ((carboxymethyl)trimethylammonium chloride hydrazide, GT) and P (1-(carboxymethyl)pyridinium chloride hydrazide, GP) were used to improve the ionization efficiency of glycans in MALDI and ESI mass spectrometry [15]. The permanent cation of Girard's reagents enhances the peak intensity in the positive-ion mode and also removes the ambiguity caused by alkali metal adducts in MALDI. On the other hand, ILMs have gained increasing attention because of their advantages in vacuum stability and signal-to-noise (S/N) ratio compared with frequently used matrices [16]. Generally, ILMs comprise matrix acid and organic base, and keep homogeneous liquid state (Scheme 1). In addition, compared with thermal reactions, microwave- (MW-) assisted tagging reaction has advantages in MALDI sample preparation, such as high coupling efficiency and short reaction time.

**Scheme 2:**
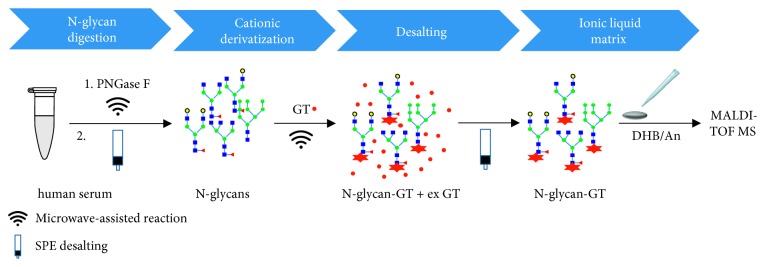
Workflow of GT-SPE-DHB/An method for N-glycan analysis in MALDI-TOF MS.

Until now, chemical derivatization and ILM approach have been individually investigated to solve the problems of MALDI-based glycan analysis. Herein, we propose a synergistic combination of chemical derivatization and ILM. A new method combining microwave-assisted Girard T derivatization, SPE desalting, and DHB/An ion liquid matrix (GT-SPE-DHB/An) was optimized and applied to the MALDI-TOF MS analysis of human serum N-glycans (Scheme 2).

## 2. Materials and Methods

### 2.1. Generals

#### 2.1.1. Materials and Reagents

Maltohexaose (47873), 2, 5-dihydroxybenzoic acid (DHB, 149357), *α*-cyano-4-hydroxycinnamic acid (CHCA, C2020), *p*-coumaric acid (*p*CA, C9008), pyridine (Pyr, 27047), horseradish peroxidase (77332), NH_4_HCO_3_ (A6141), dithiothreitol (DTT, D0632), trifluoroacetic acid (TFA, T6508) were purchased from Sigma-Aldrich. Human serum is purchased from Sigma (H4522). Triethylamine (TEA, 121448), aniline (An, A0463), 1-methylimidazole (MI, M0508), 1, 1, 3, 3- tetramethylguanidine (TMG, T0148), Girard's reagent T (GT, B0547), and Girard's reagent P (GP, G0030) were obtained from TCI. Peptide-N-glycosidase F (PNGase F, P0705L) was obtained from New England Biolabs. The N_2_ dryer SPE Dry™ 96 dual sample concentrator system was used from Biotage (SD2-9600-DHS-EU). PNGase F-released glycans were purified by graphitized carbon cartridges (GlykoClean™ Glycan Clean-up Station, Vacuum Manifold, GC 100, Prozyme). Acetonitrile was purchased from Merck (1.00030.4000). All solvents were HPLC grade.

#### 2.1.2. Instrumentation

Tinkerbell LT (ASTA Inc., Korea) was used for MALDI-TOF MS. CEM Discover™ microwave reactor was applied for the microwave-assisted reaction. Bioshaker (Bioshake iQ, Analytik Jena AG) and evaporation system (EZ-2, Genevac) or Speed-Vac concentrator (Module 4080C, Korea) were used.

### 2.2. Sample and Matrix Preparation

#### 2.2.1. Isolation of N-Linked Glycans

5 *µ*L of horseradish peroxidase (30 mg/mL) was added to each serum sample (50 *μ*L), and then 50 *μ*L of 200 mM NH_4_HCO_3_ containing 10 mM of dithiothreitol was added. Protein denaturation was carried out by placing the samples to a Bioshaker (1,500 rpm) at 65°C for 5 min. N-linked glycans were released from the denatured proteins by the addition of 1,000 units of PNGase F. The PNGase F digestion was carried out in a microwave-assisted rapid enzyme digestion system (power output = 400 W, ASTA Inc., Korea) at 37°C for 8 min. Then, ice-cold ethanol (450 *μ*L) was added, and the proteins were precipitated by centrifuging (3,700 rpm) at 4°C for 50 min. The protein precipitate was removed, and the supernatant carrying N-linked glycans was transferred to new tubes and dried by N_2_ dryer for 1 h. Then, 550 *μ*L of distilled water and 5 *μ*L of maltohexaose (25 *μ*g/mL) were added to the dried glycan samples.

#### 2.2.2. Glycan Purification

The PNGase F-released glycans were purified by solid-phase extraction of graphitized carbon cartridges (Carbograph, Alltech Associates, Inc.). Before loading, the cartridges were equilibrated with 0.1% TFA in 80% CH_3_CN and with water repeatedly. Glycan solutions (550 *μ*L) were applied to the preconditioned cartridge, and then the cartridge was washed with water subsequently. The glycans were eventually eluted in 20% CH_3_CN. Each eluted glycan sample was dried by the evaporation system at 70°C. The dried glycans were dissolved in 15 *μ*L of water to be used for derivatization and MALDI-TOF MS analysis.

#### 2.2.3. Derivatization of Maltohexaose by GT (or GP) Reagent (Thermal Condition)

A solution of GT (or GP) was prepared by dissolving 1 mg (6 *μ*mol) of the reagent in 1 mL of acetic acid/water (10:90, v/v). Maltohexaose (15 *μ*L of stock 2 mM) was added to 100 *μ*L of the solution (20-fold excess) in a screw-capped vial. The derivatization sample was heated at 75°C for 3 h. The solvents were removed by Speed-Vac. The residue was dissolved in 120 *μ*L of CH_3_CN/water (70:30, v/v), and the aliquots were analyzed directly by MALDI-TOF.

#### 2.2.4. Derivatization of N-Glycans by GT Reagent

*(1) Thermal Derivatization*. 16 *μ*L of N-glycan sample (with maltohexaose as an internal standard, IS) was mixed with 20 *μ*L of GT derivatization solution (6 mM) in acetic acid/cosolvent (15:85, v/v). The cosolvent was prepared by CH_3_OH/water (1/1, v/v). 32 *μ*L of acid acetic/cosolvent (15:85, v/v) was added to the sample. The solution was heated in Eppendorf Thermomixer (1,000 rpm, 75°C, 3 h). The solvent was evaporated, and the residue was applied to the graphitized carbon cartridge to remove excess amount of the unreacted GT. The purified, GT-tagged glycans were dried and diluted with 30 *μ*L of CH_3_CN/water (1:1, v/v).

*(2) Microwave-Assisted Derivatization*. 32 *µ*L of N-glycan sample (without spiking of IS) was mixed with 100 *µ*L of GT solution (6 mM) in acetic acid/cosolvent (20:80, v/v). The reaction was carried out in a microwave reactor (enzymatic digest mode, fiber optical thermometer, power = 60 W) at 75°C for 1 h. The solvent was removed, and the residue was purified by the graphitized carbon cartridge. After drying by Speed-Vac, the glycan derivatization sample was diluted with 60 *µ*L of CH_3_CN/water (1:1, v/v).

#### 2.2.5. Ionic Liquid Matrix Preparation

The ILM was prepared as follows: matrix (DHB 5, CHCA 6.1, *p*CA 5.4 mg, respectively) was dissolved in 1 mL of CH_3_CN/water (1:1, v/v), and base (aniline 8.8, MI 7.6, TMG 12, TEA 13.4, Pyr 7.8 *μ*L, respectively) was added to the matrix solution (1 : 3 molar ratio of matrix to base).

### 2.3. MALDI-TOF MS Measurement

MALDI-TOF mass spectra were obtained using the Tinkerbell LT instrument (ASTA Inc., Korea). The MALDI-TOF MS was operated in positive ion mode, the detector voltage (−1.9 kV), and laser power (60% for maltohexaose and 90% for N-glycans). The mass spectra were generated by 1600 laser shots. The digitizer sampling rate was at 1250 MS/sec (megasamples per second), and the full scan rate was 0.1 V for the digitizer. The mass spectra were obtained between 800 and 2400 m/z. The glycan sample was mixed with ILMs with the ratio (1 : 1, v/v), and 1.5 *μ*L was loaded on the *μ*Focus stainless steel plate (ASTA Inc., Korea). The plate was dried in a vacuum chamber for 5 h before analysis.

## 3. Results and Discussion

### 3.1. Screening of ILM Candidate and Derivatization Reagent

In the first step, 15 ILM combinations (3 matrices (DHB, CHCA, and *p*CA)) and 5 bases (An, MI, TMG, TEA, and Pyr) were prepared (ESI Figure S1) and tested with three different methods (Girard's T, P, and nonderivatization as a reference). 45 combinations (3 × 5 × 3) were screened, and maltohexaose was used as a model glycan. Among the 15 ILMs, five pairs (DHB/An, DHB/Pyr, CHCA/An, CHCA/TEA, and CHCA/MI) showed relatively high R/N ratio (Table 1) and peak intensity (ESI Table S1). Considering the ILM diversity, we selected three ILM candidates (DHB/An, CHCA/MI, and CHCA/TEA) for the next step.

The three ILM candidates and GT/GP reagents were tested with maltohexaose spiked N-glycan sample to find out the best combination of them (ESI Figure S2). GT derivatization resulted in better spectra than GP one. In addition, DHB/An pair was better than CHCA/TEA or CHCA/MI pair in the MS signal intensity and background noise. Thus, we selected GT and DHB/An combination for the further optimization. This acid/base choice agrees with Perreault's report [17], in which DHB/An ILM showed significant improvement in sensitivity for native oligosaccharides in MALDI-TOF MS.

### 3.2. Sample Preparation

For successful N-glycan MALDI-TOF MS analysis with GT-DHB/An combination, two requirements should be satisfied: complete GT coupling and high S/N ratio. Conventional parameters, such as GT equivalent, reaction temperature, and percentage of acetic acid, were taken from previous reports [15, 18] (30 equivalent of GT, 75°C, 15% acetic acid), and we checked the GT tagging efficiency under the condition. Unfortunately, the reported parameters did not work well in our N-glycan sample. To achieve complete GT coupling and high S/N ratio, we utilized microwave- (MW-) assisted GT derivatization and solid-phase extraction (SPE) desalting. Researchers have already used MW-assisted glycan derivatization [19] and desalting for the sample preparation of N-glycans [20]. The desalting process would be more important in our experiment because excess amount of the GT reagent was used.

Three conditions were tested: Condition 1 = MW-assisted GT derivatization without SPE desalting, Condition 2 = conventional GT derivatization with SPE desalting, and Condition 3 = MW-assisted GT derivatization with SPE desalting. We matched the observed MS peaks with the human serum N-linked glycome library which was reported by S. R. Kronewitter et al. [21]. A total of 13 peaks were commonly identified in the conditions. The average enhancement factors of Condition 3 were 16.6 ± 7.0 against Condition 1 and 2.1 ± 0.5 against Condition 2 (Table 2). Both MW-assisted GT derivatization and SPE desalting were essential to enhance the MS intensity on N-glycan analysis.

### 3.3. Human N-Glycan MALDI TOF MS Analysis

**Figure 1:**
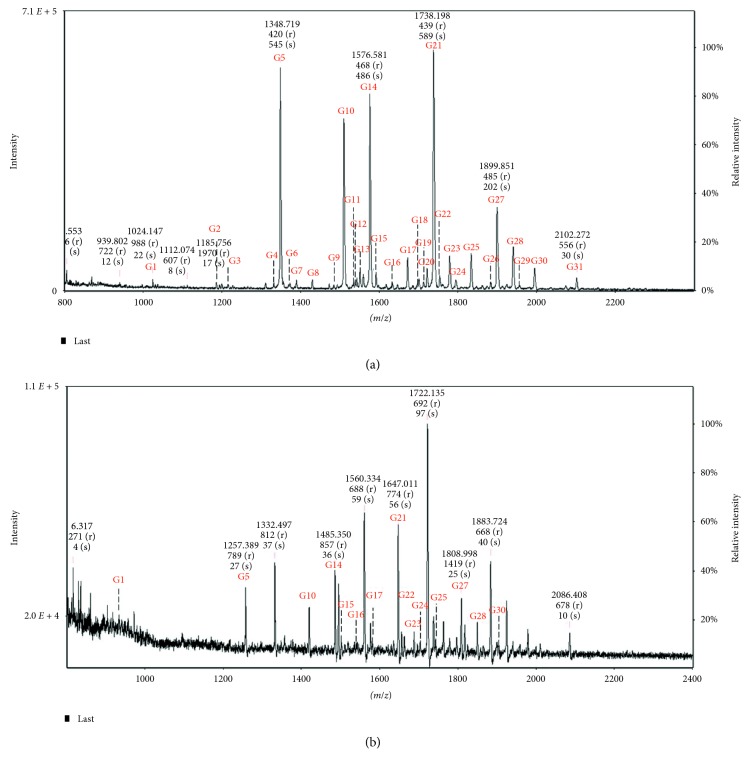
MALDI-TOF MS spectra of (a) GT derivatized N-glycans obtained from the optimized GT-SPE-DHB/An method and (b) N-glycans obtained from DHB/An method.

We were able to confirm the advantage of the combination of GT derivatization and DHB/An in the optimized MW-assisted GT derivatization and SPE desalting condition (Figure 1). Compared with the N-glycan analysis in DHB/An method, the noise level of our GT-SPE-DHB/An method was significantly reduced. The *y*-axis scale increased by approximately 7 times in our method (7.1 × 10^5^ vs. 1. 1 × 10^5^). We assigned 31 glycans using the optimized GT-SPE-DHB/An method.

In order to demonstrate the superiority of our method for N-glycan analysis, we compared the MS peak intensities of all assigned glycans by Method 1 (MW-assisted GT derivatization-SPE desalting-DHB/An ILM combination) with those by Method 2 (DHB/An ILM). Peak intensity of the assigned glycans is listed in Table 3, and the S/N ratio of them was summarized in ESI Table S2. Both peak intensity and S/N ratio showed a same trend in ranking and enhancement factor.

**Figure 2:**
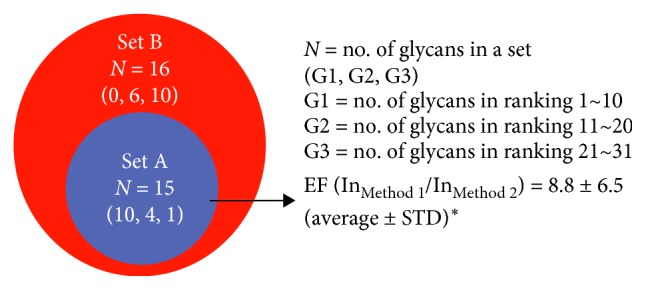
Venn diagram of the assigned glycans in Table 3. The number in the parenthesis indicates the number of assigned glycans in each ranking group. In_*i*_ = intensity of Method i in a particular peak. ^*∗*^ The average and standard deviation were calculated for the commonly assigned 15 glycans of Set A.

Thirty-one glycans were assigned by Method 1, and 15 glycans were commonly assigned by both methods. The peak intensity rankings were analyzed in the Venn diagram (Figure 2). The 31 glycans can be divided into two sets. Set A (purple colored, *N*=15) includes the glycans commonly assigned by Methods 1 and 2. Meanwhile, Set B (red colored, *N*=16), which is a complementary set of Set A, includes the glycans only assigned by Method 1. The peak intensity of a particular glycan would be related to the abundance and MALDI ionization efficiency of the glycan. All of high-ranking glycans (Group 1, ranking scale from 1 to 10) were easily assigned by both methods. However, only 4 glycans in Group 2 (ranking scale from 11 to 20) and just 1 glycan in Group 3 (ranking scale from 21 to 31) were assigned by Method 2. On the other hand, the 16 glycans in Set B belonged to Groups 2 and 3 (having low abundance and poor MALDI ionization efficiency) and were newly assigned using Method 1. Briefly, our GT-SPE-DHB/An method exhibited better sensitivity than the conventional DHB/An method.

The improved glycan sensitivity of GT-SPE-DHB/An method was also confirmed by the MS peak intensity enhancement. We calculated the enhancement factors (EFs) of 15 commonly assigned glycans in Set A. Method 1 showed 8.8 times higher peak intensity (STD ± 6.5) than Method 2. The improved EF suggests that Method 1 efficiently suppresses noise and enhances MALDI ionization compared with the DHB/An method. These data support that the glycans which have relatively low abundance and poor MALDI ionization efficiency can be detected by our GT-SPE-DHB/An method.

## 4. Conclusions

We proposed a new method combining chemical derivatization and ion liquid matrix for sensitive MALDI-TOF MS analysis of N-glycans. Our method included microwave-assisted Girard T derivatization, SPE desalting, and DHB/An ion liquid matrix (GT-SPE-DHB/An). The synergistic combination of them resulted in more sensitive glycan detection than a conventional DHB/An method in MALDI-TOF MS. Using our GT-SPE-DHB/An method, we were successfully able to assign 31 glycans from the human serum N-glycan sample in the 900–2,000 m/z range. However, with the established method, i.e., DHB/An method, only 15 glycans were assigned. To the best of our knowledge, this GT-SPE-DHB/An method is the first to combine cationic derivatization of N-glycan and ionic liquid matrix for N-glycan analysis in MALDI-TOF MS.

## Figures and Tables

**Table 1:** Red-white conditional formatting table between three variables (matrix, base, and tagging reagent) and the MALDI R/N ratio of maltohexaose or its Girard derivatives. ND = not detected. Color scale: deeper red highlighted cells correspond to higher R/N ratio.

Derivatization	Matrix	Base				
		An	MI	TMG	TEA	Pyr
Girard's T	DHB	245	5	ND	63	224
	CHCA	335	3,970	1,317	4,972	62
	*p*CA	ND	ND	ND	ND	ND

Girard's P	DHB	99	ND	ND	36	9
	CHCA	2,823	4,423	15	2,842	43
	*p*CA	ND	4	ND	ND	ND

Nonderivatization	DHB	18	ND	ND	ND	248
	CHCA	621	4,421	10	4,974	354
	*p*CA	ND	ND	ND	ND	ND

**Table 2:** Condition optimization. MS intensity for each glycan at three different conditions and the enhancement factors (EFs).^*∗*^

Glycan no.	MS intensity			Enhancement Factor	
	Condition 1 MW	Condition 2 SPE	Condition 3 MW + SPE	Con 3/Con 1 w/or w/o SPE	Con 3/Con 2 w/or w/o MW
IS	29738	299037	682825	23.0	2.3
G5	5991	72493	122582	20.5	1.7
G8	2463	9714	12278	5.0	1.3
G10	5328	56814	97849	18.4	1.7
G14	11482	91319	227387	19.8	2.5
G15	2493	11139	15844	6.4	1.4
G17	2604	10991	24068	9.2	2.2
G20	2377	31626	76822	32.3	2.4
G21	13829	93595	270122	19.5	2.9
G23	2372	20225	45111	19.0	2.2
G25	2806	20863	39855	14.2	1.9
G27	5192	32241	83983	16.2	2.6
G28	3974	22301	57710	14.5	2.6
G30	2777	20591	40422	14.6	2.0
Ave ± STD				16.6 ± 7.0	2.1 ± 0.5

^*∗*^We calculated the EFs for the glycans, which were commonly assigned at the three conditions.

**Table 3:** MALDI peak intensity table of the assigned glycans in 900–2,000 m/z range.

Glycan ID no.	MS intensity		Ranking	Group	EF
	Method 1 = GT-SPE-DHB/An	Method 2 = DHB/An			
G1	22,523	7,904	19	2	2.8
G2	14,247	—	24	3	—
G3	8,621	—	31	3	—
G4	13,752	—	25	3	—
G5	556,350	25,650	2	1	21.7
G6	12,904	—	27	3	—
G7	23,418	—	16	2	—
G8	23,395	—	17	2	—
G9	11,284	—	28	3	—
G10	428,229	18,631	4	1	23.0
G11	8,857	—	30	3	—
G12	14,250	—	23	3	—
G13	38,460	—	13	2	—
G14	495,759	33,830	3	1	14.7
G15	38,971	9,724	12	2	4.0
G16	17,100	6,714	22	3	2.5
G17	78,584	8,961	9	1	8.8
G18	13,420	—	26	3	—
G19	18,481	—	20	2	—
G20	50,126	—	11	2	—
G21	601,498	52,609	1	1	11.4
G22	30,297	10,260	15	2	3.0
G23	81,931	11,867	8	1	6.9
G24	22,814	8,421	18	2	2.7
G25	89,870	10,485	7	1	8.6
G26	18,283	—	21	3	—
G27	205,902	23,231	5	1	8.9
G28	105,748	14,170	6	1	7.5
G29	9,020	—	29	3	—
G30	52,571	8,941	10	1	5.9
G31	30,831	—	14	2	—
Ave ± STD					8.8 ± 6.5

Method 1 = GT-SPE-DHB/An method; Method 2 = DHB/An method. Group 1 = ranking scale from 1 to 10; Group 2 = from 11 to 20; Group 3 = from 21 to 31. EF = peak intensity of Method 1 ÷ peak intensity of Method 2.

## Data Availability

The data used to support the findings of this study are included within the article and the supplementary information files.

## Supplementary Materials

Supplementary Materials Each supplementary material has its own tags (Figure S1; Table S1), and the tags are included in the manuscript (e.g., ESI Table S1).
