# Supplementary material for: Combination of Microwave-Assisted Girard Derivatization with Ionic Liquid Matrix for Sensitive MALDI-TOF MS Analysis of Human Serum N-Glycans
Source: J Anal Methods Chem. 2018 Oct 21;2018:7832987. doi: 10.1155/2018/7832987 (PMC6215560; doi:10.1155/2018/7832987)
Supplement: Supplementary Materials — Each supplementary material has its own tags (Figure S1; Table S1), and the tags are included in the manuscript (e.g., ESI Table S1). [file 7832987.f1.docx]

**Electronic Supporting Information**

Combination of microwave-assisted Girard derivatization with ionic liquid matrix for sensitive MALDI-TOF MS analysis of human serum N-glycans

Hoa Thi Le,^a^ Kyu. H. Park,^b^ Woong Jung,^c^ Hyung Soon Park,^b^ Tae Woo Kim^a,*^

^a^ Graduate School of East-West Medical Science, Kyung Hee University, Yongin 17104, Republic of Korea.

^b^ R&D Center, ASTA Inc., Suwon 16229, Republic of Korea.

^c^ Department of Emergency Medicine, School of Medicine, Kyung Hee University, Seoul 02447, Republic of Korea.


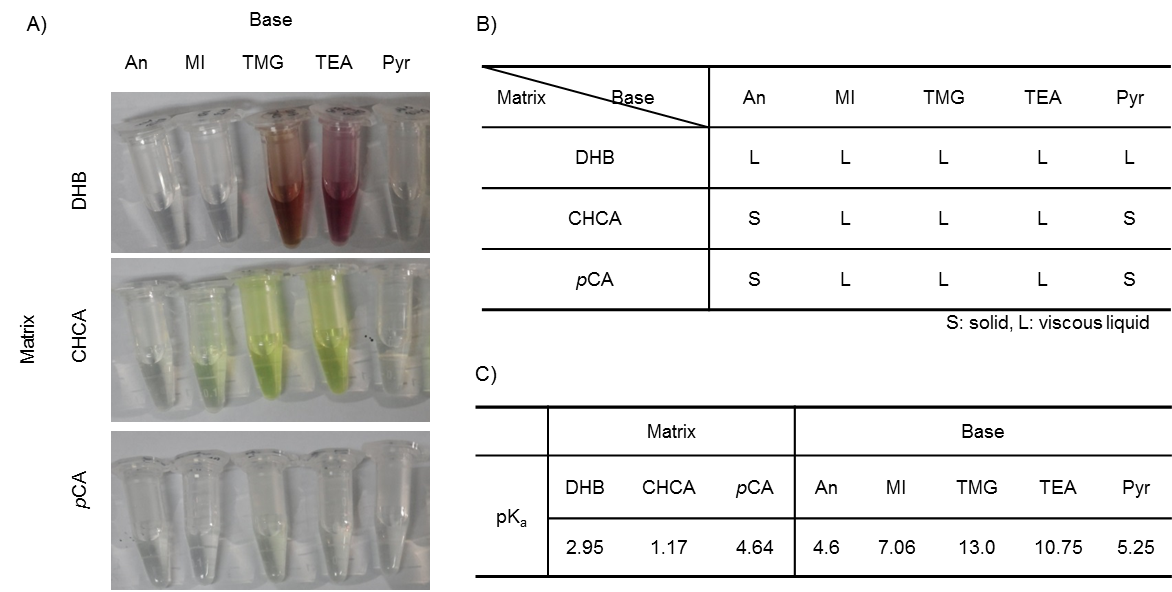


Figure S1. a) ILM colors before drying; b) ILM phase states after drying; c) pK_a_ of matrix and ammonium ion of base

Table S1. MALDI summary table for screening of ILM candidates and derivitization reagents.

| **No** | **Derivatization** | **ILM or Martix** | **Exp. *m/z*** | **Intensity** | **S/N Ratio** |
| --- | --- | --- | --- | --- | --- |
| 1 | Girard T | DHB/An | 1,104.499 | 2.60E+05 | 245 |
| 2 |  | DHB/MI | 1,104.499 | 6.31E+03 | 5 |
| 3 |  | DHB/TMG | ND | ND | ND |
| 4 |  | DHB/TEA | 1,104.540 | 8.37E+04 | 63 |
| 5 |  | DHB/Pyr | 1,104.788 | 2.92E+05 | 224 |
| 6 |  | CHCA/An | 1,104.321 | 4.48E+05 | 335 |
| 7 |  | CHCA/MI | 1,104.321 | 4.30E+06 | 3,970 |
| 8 |  | CHCA/TMG | 1,104.152 | 1.49E+06 | 1,317 |
| 9 |  | CHCA/TEA | 1,104.403 | 4.75E+06 | 4,972 |
| 10 |  | CHCA/Pyr | 1,104.260 | 8.10E+04 | 62 |
| 11 |  | *p*CA/An | ND | ND | ND |
| 12 |  | *p*CA/MI | ND | ND | ND |
| 13 |  | *p*CA/TMG | ND | ND | ND |
| 14 |  | *p*CA/TEA | ND | ND | ND |
| 15 |  | *p*CA/Pyr | ND | ND | ND |
| 16 | Girard P | DHB/An | 1,124.372 | 1.33E+05 | 99 |
| 17 |  | DHB/MI | ND | ND | ND |
| 18 |  | DHB/TMG | ND | ND | ND |
| 19 |  | DHB/TEA | 1,124.416 | 4.70E+04 | 36 |
| 20 |  | DHB/Pyr | 1,124.749 | 1.13E+04 | 9 |
| 21 |  | CHCA/An | 1,124.759 | 3.46E+06 | 2,823 |
| 22 |  | CHCA/MI | 1,124.130 | 4.54E+06 | 4,423 |
| 23 |  | CHCA/TMG | 1,124.007 | 2.01E+04 | 15 |
| 24 |  | CHCA/TEA | 1,124.171 | 3.06E+06 | 2,842 |
| 25 |  | CHCA/Pyr | 1,124.004 | 5.71E+04 | 43 |
| 26 |  | *p*CA/An | ND | ND | ND |
| 27 |  | *p*CA/MI | 1,123.133 | 3.83E+03 | 4 |
| 28 |  | *p*CA/TMG | ND | ND | ND |
| 29 |  | *p*CA/TEA | ND | ND | ND |
| 30 |  | *p*CA/Pyr | ND | ND | ND |
| continued on the next page | | | | | |

| **No** | **Derivatization** | **ILM or Martix** | **Exp. *m/z*** | **Intensity** | **S/N Ratio** |
| --- | --- | --- | --- | --- | --- |
| 31 | non-derivatization | DHB/An | 1,013.370 | 2.37E+04 | 18 |
| 32 |  | DHB/MI | ND | ND | ND |
| 33 |  | DHB/TMG | ND | ND | ND |
| 34 |  | DHB/TEA | ND | ND | ND |
| 35 |  | DHB/Pyr | 1,013.572 | 3.08E+05 | 248 |
| 36 |  | CHCA/An | 1,014.310 | 1.07E+06 | 621 |
| 37 |  | CHCA/MI | 1,013.115 | 4.72E+06 | 4,421 |
| 38 |  | CHCA/TMG | 1,013.713 | 1.47E+04 | 10 |
| 39 |  | CHCA/TEA | 1,013.183 | 5.42E+06 | 4,974 |
| 40 |  | CHCA/Pyr | 1,013.138 | 5.02E+05 | 354 |
| 41 |  | *p*CA/An | ND | ND | ND |
| 42 |  | *p*CA/MI | ND | ND | ND |
| 43 |  | *p*CA/TMG | ND | ND | ND |
| 44 |  | *p*CA/TEA | ND | ND | ND |
| 45 |  | *p*CA/Pyr | ND | ND | ND |


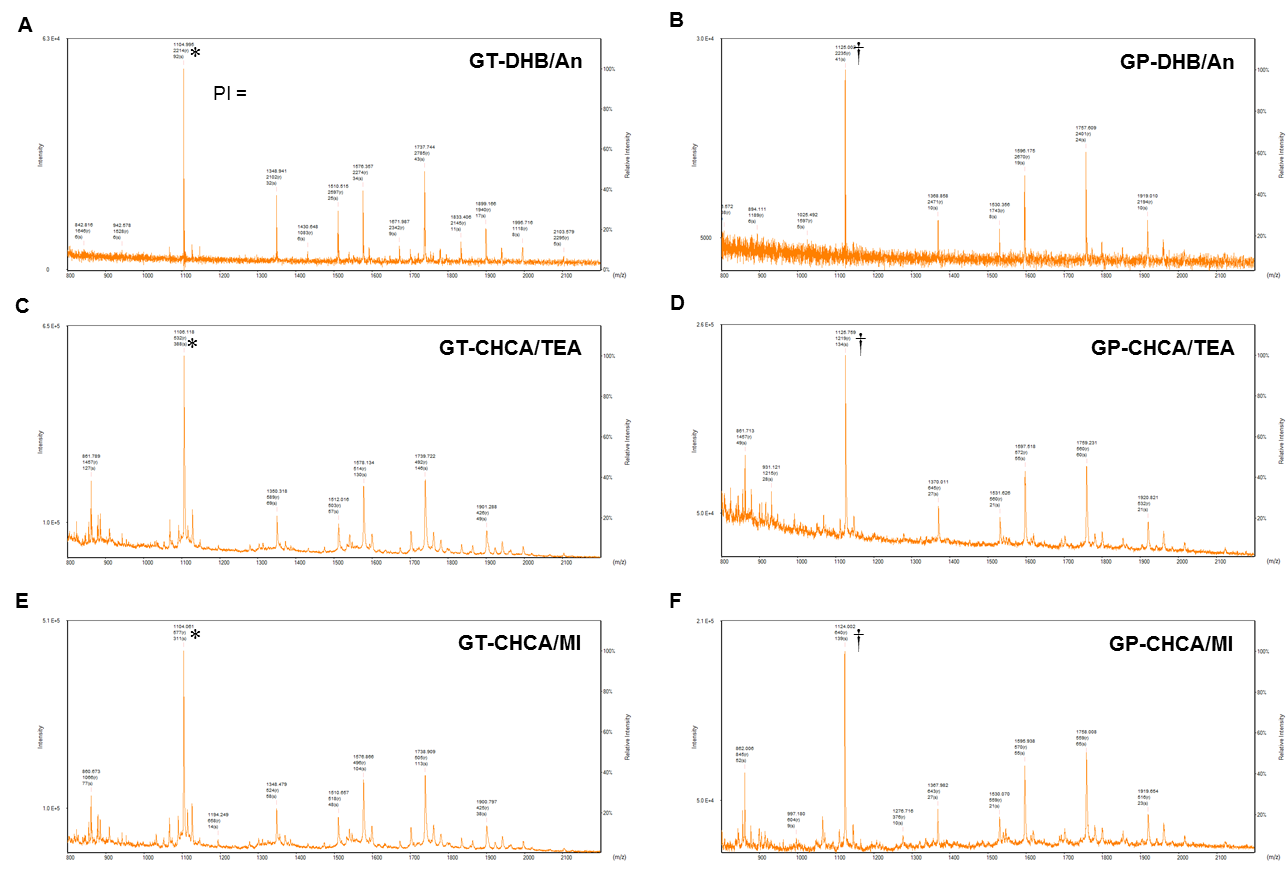


Figure S2. The MALDI MS spectra of GT or GP derivatized-glycans in three ILMs (DHB/An, CHCA/TEA, CHCA/MI). Maltohexaose (Mal, 990.327, C_36_H_62_O_31_) was spiked in the glycan sample as an internal standard. † = Mal-GT conjugate (observed m/z = 1105.06 ± 1.03, calculated m/z = 1104.43 for C_41_H_74_O_31_N_3_), ‡ = Mal-GP conjugate (observed m/z = 1124.92 ± 0.88, calculated m/z = 1124.40 for C_43_H_70_O_31_N_3_). MALDI MS condition: mass range 800-2400 *m/z*, positive ion mode, detector −1.9 kV, laser: 90%, laser shot 1200, matrix concentration 20 mg/mL.

Table S2. MALDI-TOF S/N ratio list of Method 1 and 2. Method 1 (GT-SPE-DHB/An) = the optimized method combining GT derivatization, SPE desalting, and DHB/An, Method 2 (DHB/An) = DHB/An method. The ranking follows the order of the S/N ratio of the glycans obtained by Method 1. Group 1 = ranking scale from 1 to 10; Group 2 from 11 to 20; Group 3 from 21 to 31. EF = enhancement factor of S/R ratio between Method 1 and S/R of Method 2 (AVE = 8.2, STD = ± 6.0).

| Glycan ID No. | Molecular weight | | | S/N ratio | | Ranking | Group | EF |
| --- | --- | --- | --- | --- | --- | --- | --- | --- |
|  | neat | +GT cald | +GT obsd | Method 1 GT-DHB/An | Method 2 DHB/An |  |  |  |
| G1 | 910.3 | 1024.4 | 1024.1 | 22 | 8 | 19 | 2 | 2.8 |
| G2 | 1072.4 | 1186.5 | 1186.8 | 14 | - | 23 | 3 | - |
| G3 | 1113.4 | 1227.5 | 1226.7 | 8 | - | 31 | 3 | - |
| G4 | 1218.4 | 1332.5 | 1332.4 | 13 | - | 25 | 3 | - |
| G5 | 1234.4 | 1348.5 | 1348.7 | 545 | 27 | 2 | 1 | 20.2 |
| G6 | 1259.5 | 1373.6 | 1373.7 | 13 | - | 25 | 3 | - |
| G7 | 1275.5 | 1389.6 | 1389.7 | 23 | - | 16 | 2 | - |
| G8 | 1316.5 | 1430.6 | 1430.5 | 23 | - | 16 | 2 | - |
| G9 | 1380.5 | 1494.6 | 1493.8 | 11 | - | 28 | 3 | - |
| G10 | 1396.5 | 1510.6 | 1510.5 | 420 | 20 | 4 | 1 | 21.0 |
| G11 | 1405.5 | 1519.6 | 1518.6 | 9 | - | 29 | 3 | - |
| G12 | 1421.5 | 1535.6 | 1534.9 | 14 | - | 23 | 3 | - |
| G13 | 1437.5 | 1551.6 | 1551.3 | 38 | - | 13 | 2 | - |
| G14 | 1462.5 | 1576.6 | 1576.6 | 486 | 36 | 3 | 1 | 13.5 |
| G15 | 1478.5 | 1592.6 | 1592.1 | 38 | 10 | 12 | 2 | 3.8 |
| G16 | 1519.6 | 1633.7 | 1633.1 | 17 | 7 | 22 | 3 | 2.4 |
| G17 | 1558.5 | 1672.6 | 1672 | 77 | 9 | 9 | 1 | 8.6 |
| G18 | 1583.6 | 1697.7 | 1696.4 | 13 | - | 25 | 3 | - |
| G19 | 1599.6 | 1713.7 | 1712.6 | 18 | - | 20 | 2 | - |
| G20 | 1608.6 | 1722.7 | 1721.6 | 49 | - | 11 | 2 | - |
| G21 | 1624.6 | 1738.7 | 1738.2 | 589 | 56 | 1 | 1 | 10.5 |
| G22 | 1640.6 | 1754.7 | 1753.7 | 30 | 11 | 14 | 2 | 2.7 |
| G23 | 1665.6 | 1779.7 | 1779.1 | 80 | 13 | 8 | 1 | 6.2 |
| G24 | 1681.6 | 1795.7 | 1795.1 | 22 | 9 | 18 | 2 | 2.4 |
| G25 | 1720.6 | 1834.7 | 1833.8 | 88 | 11 | 7 | 1 | 8.0 |
| G26 | 1769.6 | 1883.7 | 1883.2 | 18 | - | 20 | 2 | - |
| G27 | 1786.7 | 1900.8 | 1899.9 | 202 | 25 | 5 | 1 | 8.1 |
| G28 | 1827.7 | 1941.8 | 1940.8 | 104 | 15 | 6 | 1 | 6.9 |
| G29 | 1843.7 | 1957.8 | 1956.3 | 9 | - | 29 | 3 | - |
| G30 | 1882.6 | 1996.7 | 1995.4 | 52 | 9 | 10 | 1 | 5.8 |
| G31 | 1989.7 | 2103.8 | 2102.3 | 30 | - | 15 | 2 | - |
